# Dynamic Impact of Online Word-of-Mouth and Advertising on Supply Chain Performance

**DOI:** 10.3390/ijerph15010069

**Published:** 2018-01-04

**Authors:** Jian Feng, Bin Liu

**Affiliations:** School of Economics and Management, Shanghai Maritime University, Shanghai 201306, China; fengjian@stu.shmtu.edu.cn

**Keywords:** online word-of-mouth, advertising, supply chain performance, differential game, system dynamics

## Abstract

Cooperative (co-op) advertising investments benefit brand goodwill and further improve supply chain performance. Meanwhile, online word-of-mouth (OWOM) can also play an important role in supply chain performance. On the basis of co-op advertising, this paper considers a single supply chain structure led by a manufacturer and examines a fundamental issue concerning the impact of OWOM on supply chain performance. Firstly, by the method of differential game, this paper analyzes the dynamic impact of OWOM and advertising on supply chain performance (i.e., brand goodwill, sales, and profits) under three different supply chain decisions (i.e., only advertising, and manufacturers with and without sharing cost of OWOM with retailers). We compare and analyze the optimal strategies of advertising and OWOM under the above different supply chain decisions. Secondly, the system dynamics model is established to reflect the dynamic impact of OWOM and advertising on supply chain performance. Finally, three supply chain decisions under two scenarios, strong brand and weak brand, are analyzed through the system dynamics simulation. The results show that the input of OWOM can enhance brand goodwill and improve earnings. It further promotes the OWOM reputation and improves the supply chain performance if manufacturers share the cost of OWOM with retailers. Then, in order to eliminate the retailers from word-of-mouth fraud and establish a fair competition mechanism, the third parties (i.e., regulators or e-commerce platforms) should take appropriate punitive measures against retailers. Furthermore, the effect of OWOM on supply chain performance under a strong brand differed from those under a weak brand. Last but not least, if OWOM is improved, there would be more remarkable performance for the weak brand than that for the strong brand in the supply chain.

## 1. Introduction

Advertising, as a traditional tool of marketing communication, motivates consumer purchasing behaviors by persuasion and interest stimulation and then increases product sales. In the process of advertising, there is a cost-sharing mechanism between manufacturers and retailers, generally with manufacturers giving a certain advertising allowance to retailers. This mechanism is called cooperative (co-op) advertising [1,2,3,4]. In 2015, co-op advertising spent a total of approximately $36 billion in North America, potentially representing about 12 percent of all advertising costs [5]. Co-op advertising can enhance the whole supply chain performance by increasing sales, improving profits, and consolidating brand image [1,6]. Nowadays, due to the rapid development of the Internet, online word-of-mouth (OWOM) has become a new kind of marketing communication strategy for new products, and it is gradually attracting increasing attention from businesses. Marketing scholars have observed that word-of-mouth (WOM) referrals have a strong impact on new customer acquisition. In addition, the long-term elasticity of WOM is approximately 20 times higher than that of marketing events and 30 times higher than that of media appearances [7]. In the case of China, the proportion of online shopping customers reached 68.5% by the end of June 2017 [8]. It has also been shown that 90% of consumers read less than 10 online reviews and 58% of consumers state that the star rating of a business is the most important factor that they consider before making purchasing decisions [9]. The main factors that affect online consumers’ purchasing decisions are OWOM forms, such as online reviews or star ratings. Meanwhile, it has been reported in Europe and North America that OWOM is playing an increasingly important role in product marketing in many fields, such as in the music, mobile game, alcohol, virtual reality, and baby care industries [10,11]. Obviously, except for advertising, OWOM has deeply influenced consumers’ purchasing decisions and further affected supply chain performance (i.e., brand goodwill, sales, and profits) [12,13,14]. Therefore, it is necessary to consider the impact of co-op advertising and OWOM on supply chain performance.

There exists an extensive number of works that focus on the benefits of co-op advertising for supply chain performance. Dant and Berger (1996) analyzed co-op advertising decisions within a franchising context. The results showed that the cooperative determination of a franchiser’s and a franchisee’s advertising contributions may yield superior payoffs for whole supply chain [15]. Bergen and John (1997) introduced the competing mechanism to study the changes of participation rates of several retailers and manufacturers on co-op advertising, and its positive influence on the channel profit was also discussed [2]. Huang and Li (2001) and Li, Huang, Zhu, and Chau (2002) discussed the relationship between classical co-op advertising models and fully coordinated co-op advertising models. They also examined the effect of supply chain on the profits resulting from following coordinated strategies as opposed to leader–follower strategies [16,17]. Neyret (2009) considered the promotion of co-op advertising to supply chain profits. Then he studied the sharing mechanism between the retailer and the manufacturer on the extra joint profit achieved by moving to cooperation [18]. Liu, Cai, and Tsay (2014) evaluated the efficacy of manufacturer advertising and retailer advertising with and without cost sharing in a dual exclusive channel model with asymmetric competing supply chains. Both manufacturers’ and retailers’ cost-sharing decisions for making advertising were analyzed, respectively. Under manufacturer advertising, a lack of cost sharing with retailers was the unique equilibrium strategy for the manufacturer. However, the members in a supply chain could encounter a prisoner’s dilemma. Finally, they also evaluated the implications of advertising strategy for the whole supply chain’s efficiency [19]. Jørgensen and Gromova (2016) discussed how individual firms coordinate their advertising efforts to achieve a common objective: maximizing their overall profits. Thus, firms could try to form and maintain a cartel, the grand coalition [20]. The diffusion literature above has researched the impact of different strategies and cost-sharing ratios of co-op advertising. However, the impacts of OWOM have not been discussed.

Since online consumers spontaneously describe product features and share product experiences through online platforms, such as social networks, as well as entertainment, tourism, and shopping platforms, these forms of interactions can also motivate consumer purchasing behaviors. Considerable academic research has been generated on how OWOM affects a firm’s performance and what the relationship between OWOM and advertising is. On one hand, regarding the impact of OWOM on firm performance, Villas-Boas (2004) characterized the importance of consumer learning effects on the market outcome. Consumer demands in the second period depended on product reviews in the first period [21]. Hodac, Carson, and Moore (2013) analyzed the data from emerging and mature brands to point that the cumulative positive online customer reviews (OCRs) could increase the sales in weak brands models while the cumulative negative OCRs could decrease that. In contrast, neither positive nor negative OCRs had a significant effect on the sales in strong brands models [22]. Hu, Koh, and Reddy (2014) developed a multiple equation model to examine the inter-relationships between ratings and sales. Then they found that the ratings did not have a significant or direct impact on sales but will impact indirectly through sentiments [23]. Yu, Debo, and Kapuscinski (2016) studied the impact of consumer reviews on a firm’s dynamic pricing strategy, and they found that the firm may either enhance or dampen the information flow of the reviews via increasing or decreasing initial sales [24]. Papanastasiou and Savva (2017) analyzed product reviews and concluded that they have a significant impact on dynamic pricing of firms. They established the fact that social learning exacerbates strategic consumer behavior (i.e., increases strategic purchasing delays); its presence results in an ex ante increase in firm profit [25]. On the other hand, regarding the relationship between OWOM and advertising, Chen and Xie (2005) showed that manufacturing firms should choose advertising rather than price as a strategic variable in response to product reviews when there are enough consumers to evaluate the product’s attributes. Surprisingly, they found that, for the winning product, it could hurt the goodwill by using a review-endorsed advertising format (i.e., advertisements containing third-party award logos) to spread its positive reviews [26]. Chen and Xie (2008) also revealed that if the review information is sufficient enough, the two types of product information, i.e., the seller-created product information and the buyer-created review information, could complement or replace each other [27]. Bruce, Foutz, and Kolsarici (2012) demonstrated that in categories where new products were released in sequential stages, traditional advertising is more effective in the early stage of the product life cycle while word of mouth is more effective as consumers gain more experience with a product [28]. With the diffusion literature above, they have demonstrated a dynamic relationship between traditional advertising and OWOM. For one thing, advertising could stimulate the consumers’ online reviews [29]. For another, advertising could reduce the consumers’ WOM [30]. Nevertheless, the aforementioned literature still lacked research on the impact of OWOM on supply chain performance from the perspective of supply chains.

In summary, advertising has a direct impact on supply-chain performance, and OWOM has an indirect impact on firm’s sales via brand goodwill. Furthermore, as the product life cycle continues to evolve and move forward, there are some dynamic relationships between OWOM and advertising. However, existing research studies have rarely combined advertising and OWOM to research their dynamic impacts on supply-chain performance. Although Gopinath, Thomas, and Krishnamurthi (2014) integrated both advertising and OWOM to empirically study their impact on enterprise performance [31], there are few thorough research studies on different advertising forms, different OWOM inputs, or their dynamic relationship in supply chain. To fill this research gap, this paper considers a single supply chain structure led by the manufacturer, and examines a fundamental issue about the dynamic impact of advertising and OWOM on supply chain performance (i.e., brand goodwill, sales, and profits).

The rest of the paper is organized as follows. In Section 2, we will present an expanded Nerlove-Arrow model, demand function, and profit functions by using the differential game approach, then we will develop a system dynamics (SD) model of supply-chain brand goodwill to reflect the dynamic impact of advertising and OWOM on supply-chain performance. In Section 3, based on the Nerlove-Arrow model, we will introduce three game scenarios, such as only advertising, manufacturer without sharing the cost of OWOM with retailers, and manufacturer sharing the cost of OWOM with retailers. For each scenario, we will derive the game equilibrium solutions of all channel members. In Section 4, based on the SD model and equilibrium solutions, we will analyze three equilibrium strategies of channel members with SD simulation. Comparative analysis is also discussed in this Section. Concluding remarks and managerial implications are discussed in the last Section. All proofs of results are in Appendix A and Appendix B.

## 2. Model Development and Notations

### 2.1. The Base Model and Assumptions

The supply chain system considered in this paper consists of one manufacturer and one retailer, and the manufacturer is the channel leader. The manufacturer sells the product to the retailer while the retailer sells the product to the consumer. To improve the brand goodwill and sales, the manufacturer (*M*) usually invests in national advertising, and the retailer (*R*) invests in local advertising and OWOM. The national advertising, local advertising, and OWOM positively affect both goodwill and revenues. While the local advertising and goodwill have a positive impact on the current sales. The accumulated goodwill is supposed to summarize past and present effects of advertising and OWOM [23,32]. In our model, the accumulated goodwill will act as state variables while advertising efforts and OWOM are control variables which affect the evolution of goodwill. State and control variables also affect the profits of supply chain [20]. Generally, the manufacturer shares a part of the retailer’s cost on advertising and OWOM. The notations used in this paper are shown in Table 1.

Following the previous literature [32,33], we assume that the change of the goodwill follows the Nerlove-Arrow framework [34], i.e.,

(1) $$\overset{•}{G}(t)=\alpha A(t)+\beta B(t)+\eta W(t)-\delta G(t)$$

Due to the manufacturer’s national advertising effort, the retailer’s OWOM effort have indirect effects on the sales, while the retailer’s local advertising effort directly influence current product demand [23]. We assume the sales satisfies the following equation:

(2) S(t)=γB(t)+θG(t)

Similar to the previous literature, such as Jørgensena and Zaccour (2003) [32], the advertising cost functions are quadratic with marketing efforts, namely, $C_{M}(A)=\frac{\mu_{A}}{2}A^{2}(t)$, $C_{R}(B)=\frac{\mu_{B}}{2}B^{2}(t)$. Meanwhile, we assume the OWOM cost of the retailer as follows, $C_{R}(W)=\frac{\mu_{W}}{2}W^{2}(t)$.

Furthermore, both manufacturer and retailer have a common infinite time horizon and a common positive discount rate *λ*. Both manufacturer and retailer strive to maximize their profits. *π_M_* and *π_R_* represent the marginal profits of manufacturer and retailer, respectively. ϕ and ω mean the cost-share rate of local advertising and OWOM that the manufacturer contributes to the retailer, so the manufacturer’s share the local advertising cost is $\phi \frac{\mu_{B}}{2}B^{2}(t)$, and the OWOM cost is $\omega \frac{\mu_{W}}{2}W^{2}(t)$. Then, the manufacturer’s objective function is

(3) $$J_{M}=\int_{0}^{\infty }e^{-\lambda t}[\pi_{M}S(t)-\frac{\mu_{A}}{2}A^{2}(t)-\phi \frac{\mu_{B}}{2}B^{2}(t)-\omega \frac{\mu_{W}}{2}W^{2}(t)]dt$$

and the retailer’s

(4) $$J_{R}=\int_{0}^{\infty }e^{-\lambda t}[\pi_{R}S(t)-(1-\phi )\frac{\mu_{B}}{2}B^{2}(t)-(1-\omega )\frac{\mu_{W}}{2}W^{2}(t)]dt$$

In the following sections we will introduce three game scenarios: only advertising, manufacturer without sharing the cost of OWOM with retailers, and manufacturer’s sharing the cost of OWOM with retailers. For each scenario, we will calculate the equilibrium solutions for all channel members based on the Stackelberg game. The game proceeds as follows. Firstly, the manufacturer decides national advertising effort *A*(*t*) at time *t.* The cost-share rate of local advertising is ϕ, and the cost-share rate of OWOM is ω. Secondly, the retailer decides the local advertising effort *B*(*t*) and OWOM effort *W*(*t*) at time *t*.

### 2.2. The System Dynamics Model

**Figure 1:**
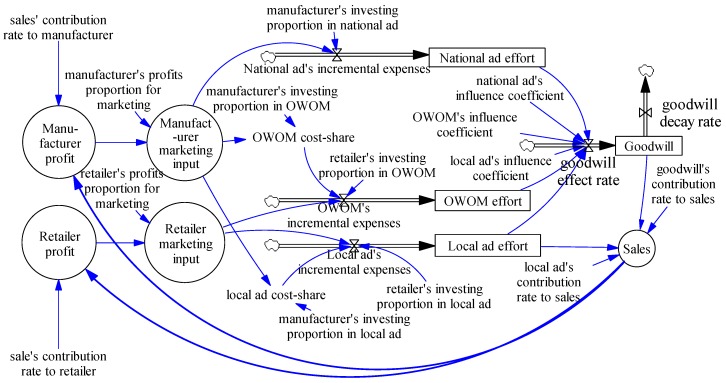
Stock and flow diagram for supply-chain goodwill.

System dynamics is a well-established methodology to model and understand the behavior of complex systems, and it has been extensively used for modeling the dynamic behavior of complex non-linear systems [35,36]. On the one hand, the national advertising, local advertising and OWOM act as input variables that positively affect the goodwill and revenues. However, the goodwill decays at the same time as it accumulates. On the other hand, the local advertising and goodwill increase the current sales and improve the supply chain profit, then lead to more investment in goodwill. The supply chain goodwill system can be dynamically recycled. To reflect the dynamic impact of advertising and OWOM on supply-chain performance, Figure 1 shows the stock and flow diagram for the system dynamics model of supply-chain goodwill by SD software-Vensim, version 7.1 (Ventana Systems, Inc., Harvard, MA, USA).

In the following sections we will simulate three scenarios (i.e., only advertising, manufacturer without sharing the cost of OWOM with retailers, and manufacturer sharing the cost of OWOM with retailers) base on this SD model.

## 3. Equilibrium Solutions

The Stackelberg equilibrium is used to analyze the supply-chain goodwill model. We obtain feedback equilibrium solutions using induction for the following three scenarios. The first scenario is the benchmark situation, where the retailer only considers investing in local advertising and the manufacturer provides co-op advertising support to the retailer. So, let *W*(*t*) = 0 in this scenario. This scenario is hereafter denoted by BM (benchmark). In the second scenario, where the retailer considers investing in both local advertising and OWOM while the manufacturer only offers a co-op advertising program to the retailer, let ω = 0. This scenario is denoted by NC (not co-op OWOM). In the third scenario, the retailer considers investing in both local advertising and OWOM. Meanwhile, the manufacturer shares both advertising costs and OWOM costs with the retailer. This scenario is denoted by C (co-op OWOM), ω∈[0,1] and *W*(*t*) > 0.

### 3.1. Only Co-op Advertising

In this scenario, consider a scenario where the manufacturer is the leader of the channel and will contribute to the retailer’s local advertising cost. Both the manufacturer and the retailer choose their strategies so as to maximize their respective profits.

**Proposition** **1.** Consider only the impact of advertising on supply chain goodwill. The equilibrium co-op advertising strategies are given by the following:

*(1) The manufacturer’s national advertising effort is*

(5) $$A^{BM}=\frac{\alpha \theta \pi_{M}}{(\delta +\lambda )\mu_{A}}$$

*and the manufacturer’s share rate for the retailer’s local advertising cost is*

(6) $$\phi^{BM}=\frac{\beta \theta \pi_{M}+\gamma (\delta +\lambda )(2\pi_{M}-\pi_{R})}{3\beta \theta \pi_{M}+\gamma (\delta +\lambda )(2\pi_{M}+\pi_{R})}$$

*(2) The retailer’s local advertising effort is*

(7) $$B^{BM}=\frac{[\beta \theta +\gamma (\delta +\lambda )][3\beta \theta \pi_{M}+\gamma (\delta +\lambda )(2\pi_{M}+\pi_{R})]\pi_{R}}{2(\delta +\lambda )\mu_{B}[\beta \theta \pi_{M}+\gamma (\delta +\lambda )\pi_{R}]}$$

*(3) The current value of the manufacturer’s profit under the equilibrium condition is*

(8) $$V_{M}^{BM}(G)=\frac{\theta \pi_{M}}{\lambda +\delta }G+\frac{\mu_{A}{\left[3\beta \theta \pi_{M}+\gamma (\delta +\lambda )(2\pi_{M}+\pi_{R})\right]}^{2}+4\theta^{2}\alpha^{2}\pi_{M}^{2}\mu_{B}}{8\lambda {\left(\delta +\lambda \right)}^{2}\mu_{A}\mu_{B}}$$

*and the retailer’s is*

(9) $$\begin{array}{ll} V_{R}^{BM}(G) & =\frac{\theta \pi_{R}}{\lambda +\delta }\cdot G+{\left\{4\lambda {\left(\delta +\lambda \right)}^{2}\mu_{A}\mu_{B}[\beta \theta \pi_{M}+\gamma (\delta +\lambda )\pi_{R}]\right\}}^{-1}\cdot \pi_{R}^{2}\\ & \times [\beta \theta^{3}(3\beta^{2}\mu_{A}+4\alpha^{2}\mu_{B})\pi_{M}+\gamma^{3}{\left(\delta +\lambda \right)}^{3}\mu_{A}(2\pi_{M}+\pi_{R})\\ & +\beta \gamma^{2}\theta {\left(\delta +\lambda \right)}^{2}(7\pi_{M}+2\pi_{R})\mu_{A}+\gamma \theta^{2}(\delta +\lambda )(4\alpha^{2}\mu_{B}\pi_{R}+\beta^{2}\mu_{A}(8\pi_{M}+\pi_{R})] \end{array}$$

For the proof, see Appendix A.

Proposition 1 illustrates the following insights. (i) There is a positive correlation between advertising efforts and marginal profits of supply chain members. (ii) The higher the product’s marginal profit is, the more expenditure on advertising and goodwill is. (iii) Co-op advertising can stimulate the market demand, enhance supply chain goodwill, and improve supply chain performance.

### 3.2. Not Co-op OWOM

In this scenario, the retailer invests in both local advertising and OWOM. Consider a game where the manufacturer is the leader of the channel. The manufacturer will contribute to the retailer’s local advertising cost, but not including the retailer’s OWOM cost. Both the manufacturer and the retailer choose their strategies so as to maximize their respective profits.

**Proposition** **2.** Consider only the impact of co-op advertising on supply chain goodwill but without co-op OWOM. Equilibrium without co-op OWOM strategies are given by:

*(1) The manufacturer’s national advertising effort is*

(10) $$A^{NC}=\frac{\alpha \theta \pi_{M}}{(\delta +\lambda )\mu_{A}}$$

*and the manufacturer’s share rate for the retailer’s local advertising cost is*

(11) $$\phi^{NC}=\frac{\beta \theta \pi_{M}+\gamma (\delta +\lambda )(2\pi_{M}-\pi_{R})}{3\beta \theta \pi_{M}+\gamma (\delta +\lambda )(2\pi_{M}+\pi_{R})}$$

*(2) The retailer’s local advertising effort is*

(12) $$B^{NC}=\frac{[\beta \theta +\gamma (\delta +\lambda )][3\beta \theta \pi_{M}+\gamma (\delta +\lambda )(2\pi_{M}+\pi_{R})]\pi_{R}}{2(\delta +\lambda )\mu_{B}[\beta \theta \pi_{M}+\gamma (\delta +\lambda )\pi_{R}]}$$

*and the retailer’s OWOM effort is*

(13) $$W^{NC}(V^{\prime })=\frac{\eta \theta \pi_{R}}{(\delta +\lambda )\mu_{W}}$$

*(3) The current value of the manufacturer’s profit under the equilibrium condition is*

(14) $$V_{M}^{NC}(G)=\frac{\theta \pi_{M}}{\lambda +\delta }G+\frac{\mu_{A}{\left[3\beta \theta \pi_{M}+\gamma (\delta +\lambda )(2\pi_{M}+\pi_{R})\right]}^{2}+4\theta^{2}\alpha^{2}\pi_{M}^{2}\mu_{B}}{8\lambda {\left(\delta +\lambda \right)}^{2}\mu_{A}\mu_{B}}+\frac{\eta^{2}\theta \pi_{M}}{(\delta +\lambda )\mu_{W}}$$

*and the retailer’s is*

(15) $$\begin{array}{ll} V_{R}^{NC}(G) & =\frac{\theta \pi_{R}}{\lambda +\delta }G+\frac{\eta^{2}\theta^{2}\pi_{R}^{2}}{2{\left(\delta +\lambda \right)}^{2}\mu_{W}}+{\left\{4\lambda {\left(\delta +\lambda \right)}^{2}\mu_{A}\mu_{B}[\beta \theta \pi_{M}+\gamma (\delta +\lambda )\pi_{R}]\right\}}^{-1}\cdot \pi_{R}^{2}\\ & \times [\beta \theta^{3}(3\beta^{2}\mu_{A}+4\alpha^{2}\mu_{B})\pi_{M}+\gamma^{3}{\left(\delta +\lambda \right)}^{3}\mu_{A}(2\pi_{M}+\pi_{R})+\beta \gamma^{2}\theta {\left(\delta +\lambda \right)}^{2}(7\pi_{M}+2\pi_{R})\mu_{A}\\ & +\gamma \theta^{2}(\delta +\lambda )(4\alpha^{2}\mu_{B}\pi_{R}+\beta^{2}\mu_{A}(8\pi_{M}+\pi_{R})] \end{array}$$

For the proof, see Appendix B.

Comparing and analyzing Propositions 1 and 2. We can find that the co-op advertising strategies are only related to the marginal profits of supply-chain members, respectively. The retailer’s OWOM effort does not change the co-op advertising strategy in the supply chain, but can increase both the manufacturer’s and the retailer’s profit. The extra profits are as follows.

The increased profit of the manufacturer as a result of the retailer’s OWOM effort is

(16) $$V_{M}^{NC}(G)-V_{M}^{BM}(G)=\frac{\eta^{2}\theta^{2}\pi_{M}^{2}}{{\left(\delta +\lambda \right)}^{2}\mu_{W}}$$

and the increased profit of the retailer by its own OWOM effort is

(17) $$V_{R}^{NC}(G)-V_{R}^{BM}(G)=\frac{\eta^{2}\theta^{2}\pi_{R}^{2}}{2{\left(\delta +\lambda \right)}^{2}\mu_{W}}$$

**Inference** **1.** The input of OWOM can improve earnings for channel members. The increasing rate of earnings is positively correlated with marginal profit and negatively correlated with the retailer’s OWOM cost coefficient.

The retailer, by Inference 1, has sufficient incentive to invest in the supply chain goodwill. The reason is that the retailer’s goodwill efforts not only improve product brand goodwill but also increase the manufacturer’s profit. That can be described in two aspects. On the positive side, the retailer can win the customer's praise and improve goodwill by promoting the service quality, such as no reason to return and customer support. On the negative side, to get good word-of-mouth, some retailers are willing to engage in word-of-mouth fraud (i.e., high praise cash back) and mislead consumers to buy. As a result, it will lead to fraud and unfair competition. In order to eliminate the retailers from word-of-mouth fraud and establish a fair competition mechanism, the third parties (i.e., regulators or e-commerce platforms) should take appropriate punitive measures against retailers. The penalty is $\frac{\eta^{2}\theta^{2}\pi_{R}^{2}}{2{\left(\delta +\lambda \right)}^{2}\mu_{W}}$.

### 3.3. Co-op OWOM

In this scenario the retailer invests in both local advertising and OWOM. Consider now a game where the manufacturer is the leader of the channel. The manufacturer will contribute to both the retailer’s local advertising costs and OWOM costs. Both the manufacturer and the retailer choose their strategies so as to maximize their respective profits.

**Proposition** **3.** Consider that the manufacturer shares both advertising costs and OWOM costs with the retailer. That is to say, the impact of both co-op advertising and co-op OWOM on supply chain goodwill should be taken into account. Equilibrium not co-op OWOM strategies are given by:

*(1) The retailer’s OWOM effort is*

(18) $$W^{C}(V^{\prime })=\frac{2\eta \theta \pi_{R}}{(\delta +\lambda )\mu_{W}}$$

*(2) The manufacturer’s share rate for the retailer’s OWOM cost is*

(19) ω=0.5

*(3) The manufacturer’s increased profit is*

(20) $$V_{M}^{C}(G)-V_{M}^{NC}(G)=\frac{2\eta^{2}\theta^{2}\pi_{M}^{2}}{{\left(\delta +\lambda \right)}^{2}\mu_{W}}-\frac{\eta^{2}\theta^{2}\pi_{R}^{2}}{4{\left(\delta +\lambda \right)}^{2}\mu_{W}}$$

*and the retailer’s is*

(21) $$V_{R}^{C}(G)-V_{R}^{NC}(G)=\frac{\eta^{2}\theta^{2}\pi_{R}^{2}}{{\left(\delta +\lambda \right)}^{2}\mu_{W}}$$

For the proof, see Appendix B (the proof process is omitted here).

Proposition 3 illustrates the following facts. (i) When the manufacturer shares half of the retailer’s OWOM cost (*ω* = 0.5), the supply chain’s OWOM effort is double. The retailer has more sufficient incentive to maintain and invest in the brand goodwill. (ii) The retailer has doubled its profit. The manufacturer's profit also increases if its profit meets the following conditions, $\pi_{M}\geq \frac{\pi_{R}}{2}$. (iii) In order to eliminate the retailers from word-of-mouth fraud and establish a fair competition mechanism, the third parties should promote the penalty to $\frac{\eta^{2}\theta^{2}\pi_{R}^{2}}{{\left(\delta +\lambda \right)}^{2}\mu_{W}}$.

## 4. System Dynamics Stimulations and Comparative Analysis

In this section, based on SD model in Section 2.2 and equilibrium solutions in Section 3, we will analyze the three equilibrium strategies of channel members by SD simulation. Comparative analysis is also conducted in this section. The three strategies are based on three game scenarios (i.e., only co-op advertising, not co-op OWOM, and co-op OWOM). Then this section tests channel members’ different efforts and cost-sharing rates in each strategy, and their impacts on supply chain goodwill and performance.

**Strategy 1.** The investment proportions of the manufacturer in national advertising and local advertising are 0.5 and 0.2, respectively. The investment proportion of the retailer in local advertising is 0.5. The manufacturer and the retailer, respectively, reserve 0.3 and 0.5 for other product marketing purposes.

**Strategy 2.** The investment proportions of the manufacturer in national advertising and local advertising are 0.5 and 0.2, respectively. The investment proportions of the retailer in local advertising and OWOM are 0.5 and 0.2, respectively. The manufacturer and the retailer, respectively, reserve 0.3 and 0.3.

**Strategy 3.** The investment proportions of the manufacturer in national advertising, local advertising, and OWOM are 0.5, 0.2, and 0.2, respectively. The investment proportions of the retailer in local advertising and OWOM are 0.5 and 0.4, respectively. Both the manufacturer and the retailer reserve 0.1.

We assume that other data are fixed. For example, the sales’ contribution rate to channel members is 50%; the profits proportion in product marketing is 30%; the decay rate of the goodwill is 5%. The stimulation figure’s horizontal axis shows the time range without specific setting (i.e., year and month), and the vertical axis is dimensionless.

By the previous literature [22,37], when brand goodwill is low enough, negative OWOM will have a great impact on it. In contrast, when brand goodwill is high enough, negative OWOM will have less impact on it. As a result, the three supply chain strategies under two scenarios, strong brand and weak brand, are analyzed through SD simulation.

### 4.1. Weak Brand Supply-Chain Goodwill Scenario

In this scenario, OWOM has a significant impact on goodwill. According to Equation (1) and the stock and flow diagram in Figure 1, we assume that positive coefficients which measure the impact of national advertising, local advertising, and OWOM on supply chain brand goodwill, are *α* = 0.4, *β* = 0.2, and *η* = 0.4, respectively. That is to say, the supply chain brand goodwill is mainly influenced by the national advertising and OWOM, then by local advertising. According to Equation (2), we assume that the positive constant *γ* = *θ* = 0.5, which represents the effect of retailer advertising and brand goodwill on sales revenue. The initial value of the supply chain weak brand goodwill is assumed to be 0.

**Figure 2:**
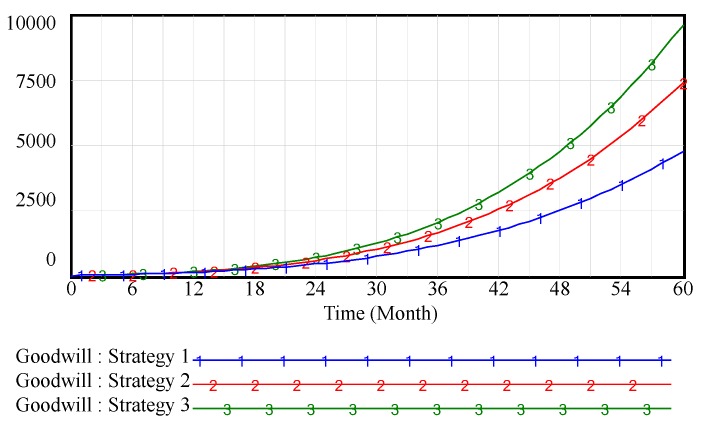
Vensim simulation results of supply-chain brand goodwill in weak brand scenario.

**Figure 3:**
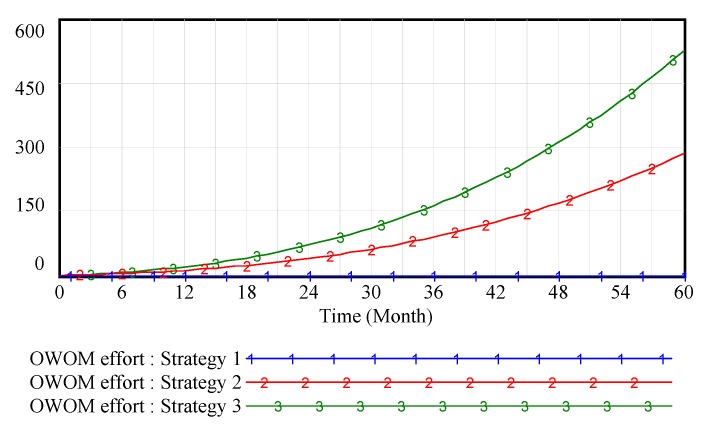
Vensim simulation results of OWOM effort in weak brand scenario.

Figure 2 shows that the cultivation of brand goodwill is a long process and requires the efforts of every channel member. In the creation stage of the brand, since the brand awareness is low, it has no significant impact on brand goodwill whether or not the channel members pay attention to inputting OWOM. With the enhancement of brand awareness, the OWOM investment of supply chain members has a significant influence on brand goodwill. OWOM has effectively enhanced the supply chain brand goodwill. Especially, Figure 3 shows that when supply chain members collaborate to engage in OWOM, it will further enhance the brand goodwill by increasing the OWOM efforts.

**Figure 4:**
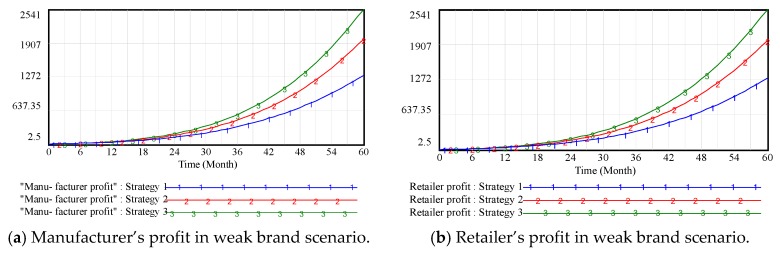
Vensim simulation results of channel members’ profits in weak brand scenario.

Figure 4 shows that brand goodwill and profit are positively correlated in the supply chain. Although the OWOM input could raise the marketing cost, the increased demand has resulted in a significant growth in profit and brand goodwill, which is similar to conclusions from Section 3.2. On the one hand, channel members have sufficient incentive to maintain the brand image of the enterprise, such as promoting the product quality, increasing customer support, and engaging in public-service activities. On the other hand, some channel members are willing to engage in word-of-mouth fraud (i.e., high praise cash back, boasting in online forums) for short-term gains but long-term OWOM losses. In order to eliminate the above adverse phenomena and establish a fair competition mechanism, the third parties should take appropriate punitive measures against supply chain members.

### 4.2. Strong Brand Supply-Chain Goodwill Scenario

In this scenario, OWOM has a less significant impact on goodwill. We assume that positive coefficients are *α* = 0.6, *β* = 0.3, *η* = 0.1, and *γ* = *θ* = 0.5 and assume the initial value of the supply-chain strong brand goodwill is 20,000.

**Figure 5:**
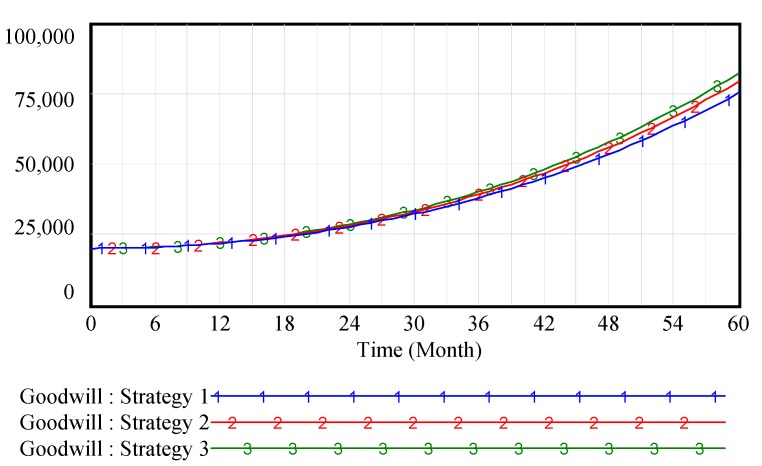
Vensim simulation results of supply-chain brand goodwill in strong brand scenario.

**Figure 6:**
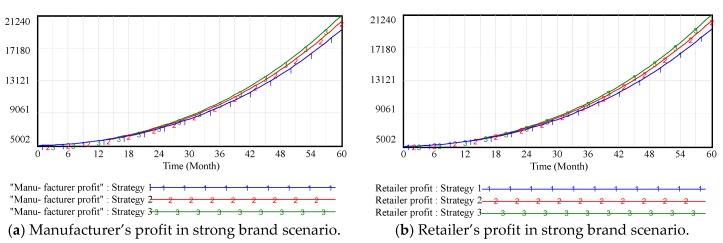
Vensim simulation results of channel members’ profits in strong brand scenario.

Figure 5 shows that when supply chain brand goodwill is high enough, it has no significant impact on brand goodwill, although channel members pay attention to OWOM input. The reason is that the product brand goodwill has accumulated to a certain level over a long period of time. OWOM has little impact on consumers’ purchasing decisions because of high brand loyalty. There is also less impact on supply chain profits, as shown in Figure 6. So, what will happen to high-value brands? The answer is that big corporate brand can bully customers! Take Toyota as an example. In 2011, it refused to recall defective automobiles in the Chinese market. The same negative phenomenon was also occurred with Samsung in 2016. It refused to recall defective mobile phones with battery explosions and lost 60% of its sales in the Chinese market. This, of course, explains why channel members should attach more importance to word-of-mouth in spite of its little significant impact on goodwill and profit. The example of Samsung’s mobile phone in China from 2016 to 2017 shows that consumers’ trust in brands will collapses in a flash while the cultivation of brand goodwill is a long process.

## 5. Conclusions

In this paper, we considered a single supply chain structure which is led by the manufacturer, and examined a fundamental issue concerning the dynamic impact of advertising and OWOM on supply-chain performance (i.e., brand goodwill, sales, and profits) under three different supply chain investment decisions. Research and analysis conclusions are showed as follows.

(i). The co-op advertising strategies are only related to marginal profits of supply chain members, respectively. The higher the product’s marginal profit is, the more cost of advertising, and also the higher the cost-sharing rate that the manufacturer accounts for. The OWOM effort cannot change the co-op advertising investing strategy in the supply chain, but it can increase both the manufacturer’s and the retailer’s profit.
(ii). The input of OWOM can improve the earnings for channel members. The retailer has sufficient incentive to invest in the supply chain goodwill. The third parties (i.e., regulators or e-commerce platforms) should take appropriate punitive measures against word-of-mouth fraud to establish a fair competition mechanism.
(iii). OWOM can not only boost goodwill but also increase profit for manufacturers. The manufacturer has sufficient incentive to share OWOM costs with its retailer. When the manufacturer shares half of the retailer’s OWOM cost, the retailer’s OWOM effort and profit are doubled. The manufacturer’s profit will also increase.
(iv). Although OWOM can effectively improve the supply chain performance, which varies by different brand goodwill, OWOM has a significant impact on a weak brand but less influence on a strong brand. As such, it proves that big corporate brand can bully customers.

The research findings above can provide instructions for how to make advertising and OWOM decisions in a supply chain from a managerial perspective. Firstly, channel members should pay great attention to and actively invest in goodwill. The goodwill input cost can be determined according to the members’ marginal profits. Secondly, there is a cost-sharing mechanism between the manufacturer and the retailer under which the manufacturer gives a certain advertising or OWOM allowance to the retailer. This mechanism can not only strengthen the supply chain co-op relationship, but also enhance the brand goodwill to improve profits. Thirdly, because the cultivation of brand goodwill is a long process, no matter how strong or weak the brand is, channel members should keep strengthening and maintaining brand goodwill.

In this paper, we studied the goodwill investment of a non-competitive supply chain; investment decisions in a competitive supply chain is a topic that will be further studied in the future.

## Figures and Tables

**Table 1:** Notations.

*t*	Time *t*, *t* ≥ 0
*G*(*t*)	The accumulated goodwill at time *t*.
*A*(*t*)	The manufacturer’s national advertising effort at time *t*.
*B*(*t*)	The retailer’s local advertising effort at time *t*.
*W*(*t*)	The retailer’s OWOM effort at time *t*.
*S*(*t*)	Sales revenue of the product along time *t*.
*C_M_*(*A*), *C_R_*(*B*)	The advertising costs of manufacturer and retailer, respectively.
*C_R_*(*W*)	The OWOM cost of the retailer.
*μ_A_, μ_B_*, *μ_W_*	Constants
*π_M_, π_R_*	Marginal profits of manufacturer and retailer, respectively.
*J_M_, J_R_*	The objective profit functions of the manufacturer and retailer, respectively.
*α* > 0	Positive coefficient measuring the impact of manufacturer advertising.
*β* > 0	Positive coefficient measuring the impact of retailer advertising.
*η* > 0	OWOM coefficient measuring the impact of the retailer OWOM.
*δ* > 0	The decay rate of the goodwill.
*γ* > 0	Positive constant representing the effect of retailer advertising on current sales revenue.
*θ* > 0	Positive constant representing the effect of goodwill on current sales revenue.
*φ* ∈ [0, 1]	Manufacturer’s share rate for the retailer’s local advertising cost.
*ω* ∈ [0, 1]	Manufacturer’s share rate for the retailer’s OWOM cost.

## Appendix A

We obtain feedback equilibrium solutions using backwards induction. We need to establish the existence of a pair of bounded and continuously differentiated value functions *V_R_*(*G*), *V_M_*(*G*) such that there exists an optimal solution *G*(*t*) to Equation (1) and the Hamilton-Jacobi-Bellman (HJB) equation,

(A1) $$\lambda V_{R}(G)=\underset{B}{\max}\{[\pi_{R}(\gamma B+\theta G)-(1-\phi )\frac{1}{2}B^{2}+V^{\prime }(G)(\alpha A+\beta B-\delta G)]\}$$

where $V^{\prime }(G)=dV_{R}(G)/dG$. Maximization of the right-hand side of the HJB equation yields,

(A2) $$B^{*}(V^{\prime })=\frac{\gamma \pi_{R}+\beta V^{\prime }}{(1-\phi )\mu_{B}}$$

The manufacturer’s profit,

(A3) $$\lambda V_{M}(G)=\underset{A,\phi }{\max}\{[\pi_{M}(\gamma B+\theta G)-\frac{\mu_{A}}{2}A^{2}(t)-\phi \frac{\mu_{B}}{2}B^{2}(t)+V^{\prime }(G)(\alpha A+\beta B-\delta G)]\}$$

Inserting Equation (A2) on Equation (A3), we get

(A4) $$A^{*}(V^{\prime })=\frac{\alpha V^{\prime }}{\mu_{A}}$$

Substituting Equations (A2) and (A4) into Equations (A1) and (A3), we simplify and obtain equation set (A5)

(A5) $$\left\{ \begin{array}{l} \lambda V_{M}(G)=\pi_{M}(\frac{\gamma^{2}\pi_{R}-\gamma \beta V^{\prime }}{(1-\phi )\mu_{B}}+\theta G)-{\frac{\phi (\gamma \pi_{R}+\beta V^{\prime })}{2{\left(1-\phi \right)}^{2}\mu_{B}}}^{2}-\frac{v^{2}\alpha^{2}}{2\mu_{a}}+V^{\prime }(G)(\frac{\alpha^{2}V^{\prime }}{\mu_{A}}+\frac{\beta \gamma \pi_{R}+\beta^{2}V^{\prime }}{(1-\phi )\mu_{B}}-\delta G)\\ \lambda V_{R}(G)=\pi_{R}(\frac{\gamma^{2}\pi_{R}-\gamma \beta V^{\prime }}{(1-\phi )\mu_{B}}+\theta G)-{\frac{\left(\gamma \pi_{R}+\beta V^{\prime }\right)}{2(1-\phi )\mu_{B}}}^{2}+V^{\prime }(G)(\frac{\alpha^{2}V^{\prime }}{\mu_{A}}+\frac{\beta \gamma \pi_{R}+\beta^{2}V^{\prime }}{(1-\phi )\mu_{B}}-\delta G) \end{array}\right.$$

We shall show that linear optimal value functions satisfy equation set (A5). Hence, we define

(A6) $$V_{M}(G)=e_{1}G+e_{2}$$

(A7) $$V_{R}(G)=s_{1}G+s_{2}$$

where *e*_1_, *e*_2_, *s*_1_, and *s*_2_ are constants.

By using the method of undetermined coefficients, we obtain

(A8) $$V_{M}(G)=e_{1}G+\frac{\left(e_{1}\beta +\gamma \pi_{R})(e_{1}\beta +\gamma \pi_{M}\right)}{\lambda \mu_{B}(1-\phi )}-\frac{e_{1}{}^{2}\alpha^{2}}{\lambda \mu_{A}}+\frac{{\left(e_{1}\beta +\gamma \pi_{R}\right)}^{2}\phi }{2\mu_{B}{\left(1-\phi \right)}^{2}}$$

where,

(A9) $$e_{1}=\frac{\theta \pi_{M}}{\lambda +\delta }$$

We use first-order maximization and get $\phi^{*}$, such as Equation (6).

Substituting Equation (6) into equation set (A5), we obtain

(A10) $$e_{2}=\frac{\mu_{A}{\left[3\beta \theta \pi_{M}+\gamma (\delta +\lambda )(2\pi_{M}+\pi_{R})\right]}^{2}+4\theta^{2}\alpha^{2}\pi_{M}^{2}\mu_{B}}{8\lambda {\left(\delta +\lambda \right)}^{2}\mu_{A}\mu_{B}}$$

(A11) $$s_{1}^{}=\frac{\theta \pi_{R}}{\lambda +\delta }$$

(A12) $$\begin{array}{ll} s_{2} & ={\left\{4\lambda {\left(\delta +\lambda \right)}^{2}\mu_{A}\mu_{B}[\beta \theta \pi_{M}+\gamma (\delta +\lambda )\pi_{R}]\right\}}^{-1}\cdot \pi_{R}^{2}\cdot [\beta \theta^{3}(3\beta^{2}\mu_{A}+4\alpha^{2}\mu_{B})\pi_{M}\\ & +\gamma^{3}{\left(\delta +\lambda \right)}^{3}\mu_{A}(2\pi_{M}+\pi_{R})+\beta \gamma^{2}\theta {\left(\delta +\lambda \right)}^{2}(7\pi_{M}+2\pi_{R})\mu_{A}\\ & +\gamma \theta^{2}(\delta +\lambda )(4\alpha^{2}\mu_{B}\pi_{R}+\beta^{2}\mu_{A}(8\pi_{M}+\pi_{R})] \end{array}$$

Substituting Equations (A9)–(A12) into Equations (A6) and (A7), we get Equations (8) and (9). Then substituting Equation (A9) into Equations (A2) and (A4), we also obtain Equations (5) and (7).

Proof finished.

## Appendix B

The proof process is the same as in Appendix A. We use the HJB equation,

(A13) $$\lambda V_{R}(G)=\underset{B,W}{\max}\{[\pi_{R}(\gamma B+\theta G)-(1-\phi )\frac{1}{2}B^{2}-\frac{\mu_{W}}{2}W^{2}(t)+V^{\prime }(G)(\alpha A+\beta B+\eta W-\delta G)]\}$$

(A14) $$B^{*}(V^{\prime })=\frac{\gamma \pi_{R}+\beta V^{\prime }}{(1-\phi )\mu_{B}}$$

(A15) $$W^{*}(V^{\prime })=\frac{\eta V^{\prime }}{\mu_{W}}$$

(A16) $$\lambda V_{M}(G)=\underset{A,\phi }{\max}\{[\pi_{M}(\gamma B+\theta G)-\frac{\mu_{A}}{2}A^{2}(t)-\phi \frac{\mu_{B}}{2}B^{2}(t)+V^{\prime }(G)(\alpha A+\beta B+\eta W-\delta G)]\}$$

(A17) $$A^{*}(V^{\prime })=\frac{\alpha V^{\prime }}{\mu_{A}}$$

(A18) $$\left\{ \begin{array}{l} \lambda V_{M}(G)=\pi_{M}(\frac{\gamma^{2}\pi_{R}-\gamma \beta V^{\prime }}{(1-\phi )\mu_{B}}+\theta G)-{\frac{\phi (\gamma \pi_{R}+\beta V^{\prime })}{2{\left(1-\phi \right)}^{2}\mu_{B}}}^{2}-\frac{v^{2}\alpha^{2}}{2\mu_{a}}+V^{\prime }(G)(\frac{\alpha^{2}V^{\prime }}{\mu_{A}}+\frac{\beta \gamma \pi_{R}+\beta^{2}V^{\prime }}{(1-\phi )\mu_{B}}+\frac{\eta^{2}V^{\prime }}{\mu_{W}}-\delta G)\\ \lambda V_{R}(G)=\pi_{R}(\frac{\gamma^{2}\pi_{R}-\gamma \beta V^{\prime }}{(1-\phi )\mu_{B}}+\theta G)-{\frac{\left(\gamma \pi_{R}+\beta V^{\prime }\right)}{2(1-\phi )\mu_{B}}}^{2}+V^{\prime }(G)(\frac{\alpha^{2}V^{\prime }}{\mu_{A}}+\frac{\beta \gamma \pi_{R}+\beta^{2}V^{\prime }}{(1-\phi )\mu_{B}}+\frac{\eta^{2}V^{\prime }}{\mu_{W}}-\delta G) \end{array}\right.$$

(A19) $$V_{M}(G)=e_{3}G+e_{4}$$

(A20) $$V_{R}(G)=s_{3}G+s_{4}$$

where *e*_3_, *e*_4_, *s*_3_, and *s*_4_ are constants.

(A21) $$\begin{array}{ll} V_{M}(G)= & e_{3}G-\frac{\alpha^{2}\theta^{2}\pi_{M}{}^{2}}{2{\left(\delta +\lambda \right)}^{2}\mu_{A}}+\frac{\theta \pi_{M}}{\delta +\lambda }[\frac{\alpha^{2}\theta \pi_{M}}{(\delta +\lambda )\mu_{A}}+\frac{\eta^{2}\theta \pi_{M}}{(\delta +\lambda )\mu_{W}}+\frac{\beta }{\mu_{B}(1-\phi )}(\frac{\beta \theta \pi_{M}}{\delta +\lambda }+\gamma \pi_{R})]\\ & +\frac{\beta }{\mu_{B}(1-\phi )}(\frac{\beta \theta \pi_{M}}{\delta +\lambda }+\gamma \pi_{R}-\frac{\phi }{2\mu_{B}{\left(1-\phi \right)}^{2}}{\left(\frac{\beta \theta \pi_{M}}{\delta +\lambda }+\gamma \pi_{R}\right)}^{2} \end{array}$$

(A22) $$e_{3}=\frac{\theta \pi_{M}}{\lambda +\delta }$$

(A23) $$\begin{array}{ll} e_{4} & =\frac{v\eta^{2}\theta \pi_{R}}{(\delta +\lambda )\mu_{W}}-\frac{v^{2}\eta^{2}}{2\mu_{W}}+{\left\{4\lambda {\left(\delta +\lambda \right)}^{2}\mu_{A}\mu_{B}[\beta \theta \pi_{M}+\gamma (\delta +\lambda )\pi_{R}]\right\}}^{-1}\cdot \pi_{R}^{2}\\ & \times [\beta \theta^{3}(3\beta^{2}\mu_{A}+4\alpha^{2}\mu_{B})\pi_{M}+\gamma^{3}{\left(\delta +\lambda \right)}^{3}\mu_{A}(2\pi_{M}+\pi_{R})\\ & +\beta \gamma^{2}\theta {\left(\delta +\lambda \right)}^{2}(7\pi_{M}+2\pi_{R})\mu_{A}+\gamma \theta^{2}(\delta +\lambda )(4\alpha^{2}\mu_{B}\pi_{R}+\beta^{2}\mu_{A}(8\pi_{M}+\pi_{R})] \end{array}$$

(A24) $$s_{3}^{}=\frac{\theta \pi_{R}}{\lambda +\delta }$$

(A25) $$\begin{array}{ll} s_{4} & =\frac{\eta^{2}\theta^{2}\pi_{R}^{2}}{2{\left(\delta +\lambda \right)}^{2}\mu_{W}}+{\left\{4\lambda {\left(\delta +\lambda \right)}^{2}\mu_{A}\mu_{B}[\beta \theta \pi_{M}+\gamma (\delta +\lambda )\pi_{R}]\right\}}^{-1}\cdot \pi_{R}^{2}\\ & \times [\beta \theta^{3}(3\beta^{2}\mu_{A}+4\alpha^{2}\mu_{B})\pi_{M}+\gamma^{3}{\left(\delta +\lambda \right)}^{3}\mu_{A}(2\pi_{M}+\pi_{R})\\ & +\beta \gamma^{2}\theta {\left(\delta +\lambda \right)}^{2}(7\pi_{M}+2\pi_{R})\mu_{A}+\gamma \theta^{2}(\delta +\lambda )(4\alpha^{2}\mu_{B}\pi_{R}+\beta^{2}\mu_{A}(8\pi_{M}+\pi_{R})] \end{array}$$

Substituting Equations (A22)–(A25) into Equations (A19) and (A20), we get Equations (14) and (15). Then substituting Equation (A22) into Equations (A14)–(A16), we also obtain Equations (10), (12), and (13).

Proof finished.
